# Unveiling new horizons in severe aplastic anemia management: a two-decade study on intensive immunosuppressive therapy combined with unrelated cord blood efficacy

**DOI:** 10.3389/fimmu.2025.1622326

**Published:** 2025-07-18

**Authors:** Zhipeng Li, Xiaolin Yu, Xiaochen Song, Wenjun Li, Lei Deng, Fanjun Kong, Jing Wang, Meiling Ni, Fang Zhou

**Affiliations:** ^1^ The 960th Hospital of The People’s Liberation Army (PLA), Joint Logistics Support Force, Jinan, China; ^2^ School of Clinical Medicine, Shandong Second Medical University, Weifang, China

**Keywords:** unrelated cord blood, severe aplastic anemia, immunosuppressive therapy, SAA, chimerism

## Abstract

**Background:**

In the absence of a human leukocyte antigen (HLA)-matched donor, intensive immunosuppressive therapy (IST) combined with unrelated cord blood (IIST-UCB) a salvage treatment option for patients with severe aplastic anemia (SAA) who had failed IST. With advancements in transplantation technology, outcomes of IIST-UCB have improved considerably in recent years. Here, we will focus on the differential effects of IIST-UCB on patient survival and GVHD risk and evaluate its therapeutic efficacy between SAA and VSAA patients.

**Methods:**

Between August 2004 and May 2024, 115 SAA patients were screened at enrollment. The overall survival (OS) rates and failure-free survival (FFS) rates were evaluated and compared using Kaplan–Meier curves and log-rank tests. Cumulative incidences of cytomegalovirus (CMV), hematopoietic recovery, and Epstein–Barr virus (EBV) were estimated using a competing risk regression model.

**Results:**

The median age was 16 years (range, 2–74). At 6 months, 27 patients (27%) achieved complete response (CR), and 44 patients (44%) achieved partial response (PR). The median period to neutrophil engraftment was 25 days, and to platelet engraftment was 44 days. The 250-day cumulative incidence of hemoglobin recovery was 87.8% (95% CI, 77.7%–93.6%). The 100-day cumulative incidence of neutrophil engraftment was 88.5% (95%CI, 80.6%–93.3%). The 400-day cumulative incidences of platelet engraftment was 86.7% (95%CI, 77.5%–92.4%). The 5-year overall survival was 86.1% ± 6.66%, and the 5-year failure-free survival was 72% ± 8.62% in the cohort. Transplantation-related mortality was 12.5% (95% CI, 7.2%–19.4%). No acute or chronic graft-versus-host disease (GVHD) was observed during the entire period. The cumulative incidences of CMV and EBV were 7.18% (95% CI, 3.34%–13%) and 16.8 (95% CI, 10.6%–24.3%), respectively. The majority of patients exhibited microchimerism and maintained hematopoiesis over the long term. Patients with SAA who received UCB treatment showed significantly higher hematopoietic reconstitution efficiency (*P =* 0.004, *P =* 0.001, *P =* 0.001) and overall survival compared with the VSAA group (*P =* 0.028).

**Conclusion:**

These data support IIST-UCB as an alternative therapeutic approach for patients with SAA.

## Introduction

Severe aplastic anemia (SAA), a life-threatening condition, is characterized by pancytopenia and hypocellular bone marrow (1, 2). A matched sibling donor (MSD) remains a cornerstone of curative therapy for SAA, offering high survival rates for SAA patients. In parallel, immunosuppressive therapy (IST) is a modern frontline treatment for SAA (3). For patients who fail IST or lack a matched sibling, unrelated donor (URD) transplants, unrelated cord blood (UCB), and haploidentical hematopoietic stem cell transplantation (haplo-HSCT) may serves as treatment options. Identifying a URD can be time-consuming, potentially causing patients to miss the optimal window for therapy. Recent advances in alternative donor transplantation, particularly with haploidentical donors, have expanded treatment options for patients with SAA. Haploidentical HSCT has demonstrated remarkable improvements in survival and quality of life, owing to enhanced graft manipulation techniques, reduced-intensity conditioning regimens, and effective graft-versus-host disease (GVHD) prophylaxis (4, 5). Despite these advances, challenges persist for patients lacking timely access to an MSD or haploidentical donor. Thus, seeking alternative treatments remains necessary. Unrelated cord blood (UCB) has been used to treat SAA due to its rapid availability and low risk of GVHD (6–8).

As previously reported, de Latour confirmed the efficacy and safety of UCB in patients with SAA (9). Our previous study demonstrated the efficacy of intensive IST combined with UCB (IIST-UCB) in children with SAA, with the IIST-UCB group showing a significantly higher overall response (OR) than the IST group (10, 11). Here, we will focus on the differential effects of IIST-UCB on patient survival and the risk of GVHD and evaluate its therapeutic efficacy between patients with SAA and patients with very SAA (VSAA).

## Methods

### Patients

The study included patients who met the inclusion criteria. The inclusion criteria were (1): confirmed diagnosis of SAA or VSAA (3); and (2) receipt of IIST-UCB. The Ethics Committee of the 960th Hospital of the People’s Liberation Army approved the study.

### Treatment protocols

Patients with SAA were treated with rabbit antithymocyte globulin (ATG) (3 mg/kg/day, from −6 days to −2 days), cyclophosphamide (CTX) (50 mg/kg/day, from −3 days to −2 days), and received UCB infusion on day 0. Intravenously, patients were given 3 mg/kg daily of cyclosporine A (CsA) starting on day −1 until they were able to transition to oral administration. CsA dosage was adjusted based on blood concentrations, maintained between 150 ng/mL and 250 ng/mL. The UCB was provided by the Shandong Cord Blood Bank. All patients received ganciclovir prophylaxis (250 mg every 12 h, adjusted for renal function) for 7 days before HSCT. Cytomegalovirus (CMV) and Epstein–Barr virus (EBV) DNA levels were monitored weekly during hospitalization (lower limits of detection: 500 copies/mL for CMV, 5 ∗ 103 copies/mL for EBV). Treatment protocols and diagnostic criteria were applied consistently throughout the study period. Supportive care followed contemporaneous guidelines and did not alter the core IIST-UCB protocol.

### Chimerism measurements

The chimerism assay was performed as previously reported (12). Chimerism was also assessed on days 30, 60, and 90. Complete chimerism was defined as >95% donor-derived cells. Mixed chimerism was defined as 5%–95% donor-derived cells, and microchimerism as <5% donor-derived cells.

### Definitions

Neutrophil engraftment was defined as three consecutive days with an absolute neutrophil count (ANC) >0.5 × 10^9^/L. Platelet (PLT) engraftment was defined as the first of three consecutive days with PLT >20 × 10^9^/L, without transfusion support for at least 7 days. Primary graft failure was defined as failure of neutrophils engraftment by day 42, and secondary graft failure as a decline in ANC to <0.5 × 10^9^/L after initial recovery (13, 14). Complete response (CR) was defined as ANC >1.5 × 10^9^/L, hemoglobin >100 g/L, and PLT >100 × 10^9^/L. Partial response (PR) was defined as not meeting SAA/VSAA criteria and no longer requiring transfusion support. No response (NR) was defined as continued transfusion dependence (15). Overall response (OR) was defined as the sum of CR and PR. Overall survival (OS) was defined as the time from transplant to death. Failure-free survival (FFS) was defined as the time from transplantation to the occurrence of primary graft failure, secondary graft failure, death, or relapse. Relapse was defined as disease recurrence. Transplantation-related mortality (TRM) was defined as death attributable to transplantation rather than SAA relapse.

### Statistical analysis

The chi-square test and Wilcoxon test were used for categorical and continuous variables, respectively. Kaplan–Meier curves and log-rank tests were used to evaluate and compare OS and FFS, respectively. Cumulative incidences of CMV, hematopoietic recovery, and EBV were estimated using a competing risk regression model, with death considered a competing event. Statistical analyses were performed using R version 4.4.1 or SPSS version 26. A two-tailed P-value < 0.05 was considered statistically significant.

## Results

### Patient characteristics and treatment outcome

Between 2004 and 2024, 115 patients with SAA were screened at enrollment. **Table 1** presents the baseline demographic and disease characteristics of patients with SAA. Treatment and outcome are summarized in **Table 2**.

**Table 1:** Clinical characteristics of patients.

Characteristic	IIST-UCB (N=115)
Median age, years (range)	16 (2–74)
Age, years, n (%)	
≤20 years	72 (62.61%)
20–40years	30 (26.09%)
≥40 years	13 (11.3%)
Patient sex, n (%)	
Male	66 (57.39%)
Female	49 (42.61%)
Severity of disease	
SAA	67 (58.26%)
VSAA	48 (41.74%)
SAA with PNH clone, n (%)	7 (6.09%)
Median cord TNC, ×10^8^/kg (range)	17.47 (10.38–26.41)
Median cord CD34+ cells, ×10^6^/kg (range)	6.75 (0.96–19.91)

IIST-UCB, intensive immunosuppressive therapy combined with umbilical cord blood; SAA, severe aplastic anemia; PNH, paroxysmal nocturnal hemoglobinuria; VSAA, very severe aplastic anemia; TNC, total nuclear cells.

**Table 2:** Clinical outcomes after HSCT.

Characteristic	IIST-UCB (N=115)
Primary graft failure	15 (13.04%)
Secondary graft failure	1 (0.87%)
Infection, n (%)	
Pulmonary infections	45 (39.13%)
Septicemia	20 (17.39%)
Hemorrhagic cystitis	6 (5.22%)
Febrile neutropenia	34 (29.57%)
Soft tissue infection	8 (6.96%)
Follow-up, months, median (range)	23.9 (0.73–163.83)

### Graft failure, efficacy, and hematopoietic recovery

**Figure 1:**
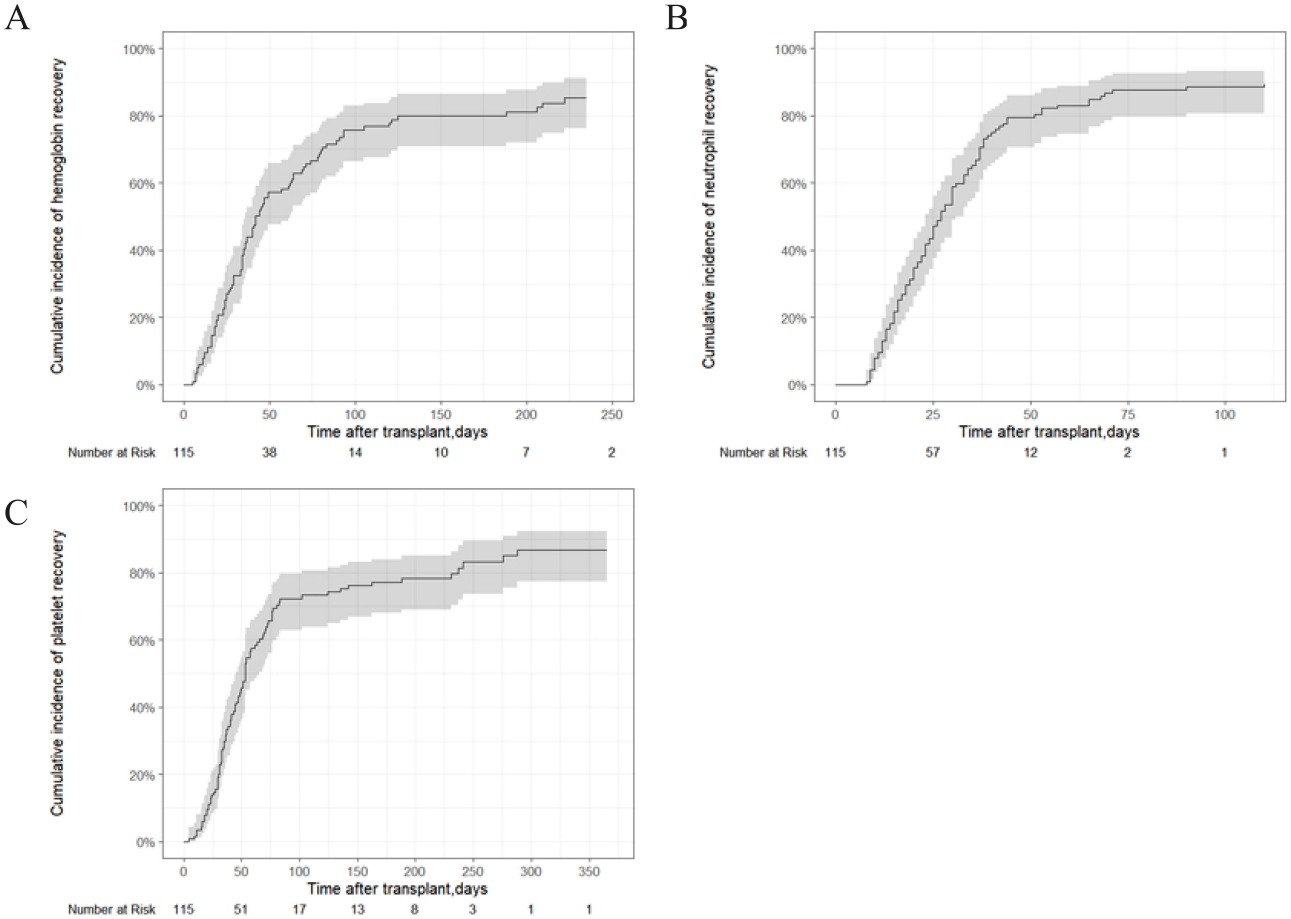
Hematopoietic recovery of patients with SAA who underwent IIST-UCB. **(A)** Cumulative incidence of Hb recovery; **(B)** Cumulative incidence of neutrophil recovery; **(C)** Cumulative incidence of neutrophil recovery.

Primary graft failure occurred in 15 patients (13.04%), all of whom received granulocyte colony-stimulating factor (G-CSF) therapy, and six additionally received mesenchymal stem cell (MSC) treatment. Of these, 14 patients achieved successful engraftment, while one patient ultimately succumbed to complications. Secondary graft failure occurred in one patient (0.87%), who experienced graft rejection 323 days post-transplantation (**Table 1**). At the 3-month follow-up, 107 patients participated in the efficacy evaluation, and 100 patients were evaluated at 6 months. After 3 months, 15 patients (14.02%) achieved CR, and 54 patients (50.47%) achieved PR. The overall response (OR) rate for IIST-UCB was 69 patients (64.49%). After 6 months, 27 patients (27%) achieved CR and 44 patients (44%) achieved PR. The overall response (OR) rate for IIST-UCB was 71 patients (71%). During the follow-up, 101 patients (87.83%) achieved neutrophil engraftment, and 93 patients (80.87%) achieved PLT engraftment. The median time to neutrophil engraftment was 25 days (range, 8–110), while time to PLT engraftment was 44 days (range, 4–288). The 250-day cumulative incidence of hemoglobin (Hb) recovery was 87.8% (95% CI, 77.7%–93.6%) (**Figure 1A**). The 100-day cumulative incidence of neutrophil engraftment was 88.5% (95%CI, 80.6%–93.3%) (**Figure 1B**). The 400-day cumulative incidence of PLT engraftment was 86.7% (95%CI, 77.5%–92.4%) (**Figure 1C**).

### OS, FFS, TRM, GVHD, and viral infectious complications

**Figure 2:**
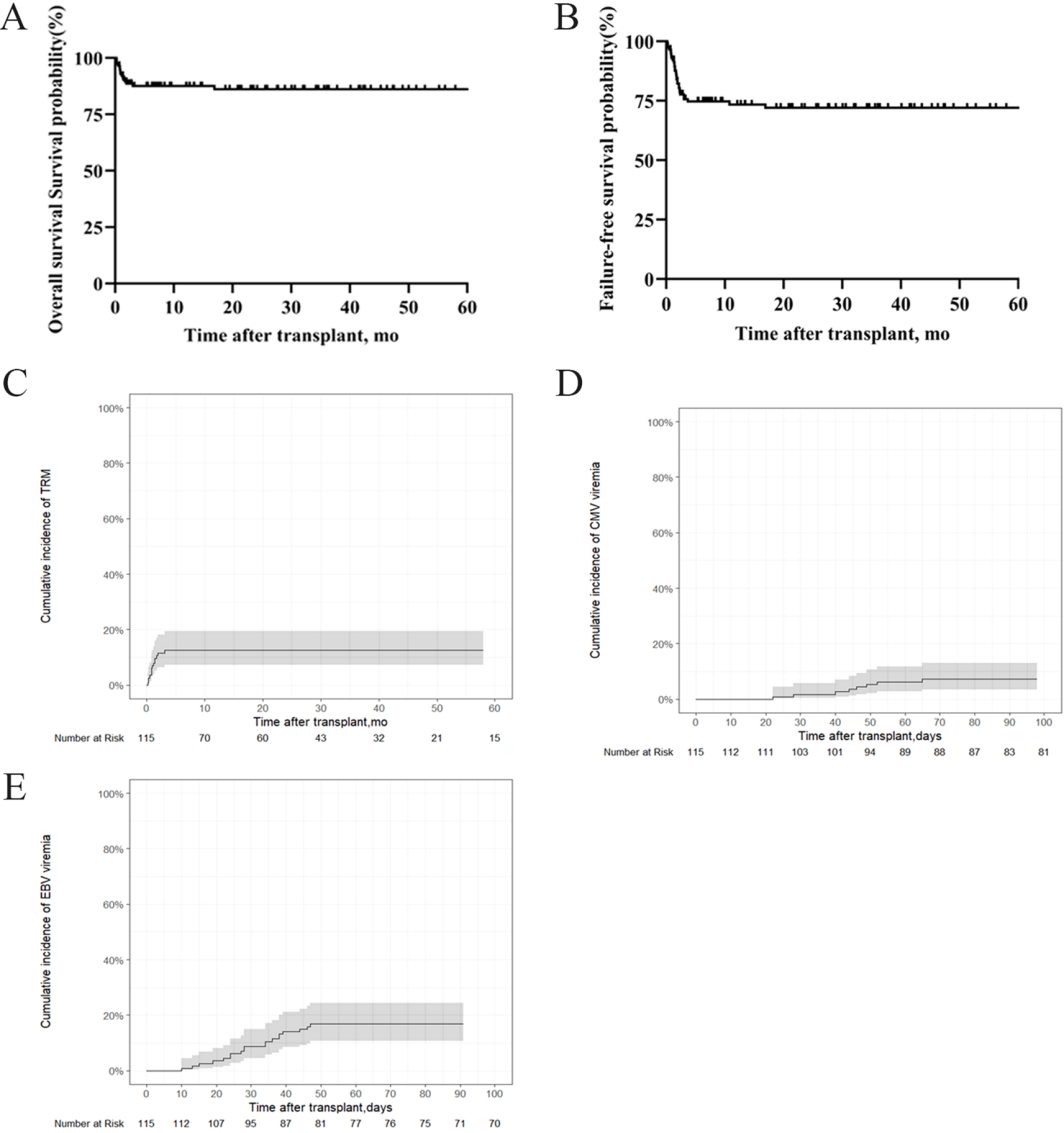
OS, FFS, TRM and infectious complications in patients with SAA who underwent IIST-UCB. **(A)** OS; **(B)** FFS; **(C)** TRM; **(D)** Cumulative incidence of cytomegalovirus (CMV); **(E)** Epstein-Barr virus (EBV).

The 5-year OS rate was 86.1% ± 6.66%, and the 5-year FFS rate was 72% ± 8.62% in the entire cohort (**Figures 2A, B**). The TRM was 12.5% (95% CI, 7.2%–19.4%) (**Figure 2C**). No GVHD was observed over a whole period. The cumulative incidences of CMV and EBV viremia were 7.18% (95%CI, 3.34%–13%) and 16.8 (95%CI, 10.6%–24.3%), respectively (**Figures 2D, E**).

### Chimerism measurements

The chimerism status of patients with SAA is summarized in **Table 3**. Bone marrow chimerism was measured in 29 patients, with 18 showing T-cell chimerism in the bone marrow, 23 showing peripheral blood chimerism, and 18 showing T-cell chimerism in the peripheral blood. Over time, the frequency of microchimerism increased. Two cases of mixed chimerism converted to microchimerism at 136 and 342 days post-transplantation, respectively.

**Table 3:** Chimerism measurements.

Measurement time	Bone marrow chimerism (n = 29)		T-cell chimerism in Bone marrow (n = 18)		Peripheral blood chimerism (n = 23)		T-cell chimerism in peripheral blood (n = 18)	
	<5% Donor	5%–95% Donor	<5% Donor	5%–95% Donor	<5% Donor	5%–95% Donor	<5% Donor	5%–95% Donor
Day 30	6 (33.3%)	12 (66.7%)	6 (50%)	6 (50%)	3 (42.9%)	4 (57.1%)	2 (50%)	2 (50%)
Day 60	11 (91.7%)	1 (8.3%)	8 (100%)	0	9 (69.2%)	4 (30.8%)	4 (57.1%)	3 (42.9%)
Day 90	3 (75%)	1 (25%)	1 (50%)	1 (50%)	3 (50%)	3 (50%)	3 (75%)	1 (25%)

### Hematopoietic recovery and survival analysis of the SAA and VSAA groups

**Figure 3:**
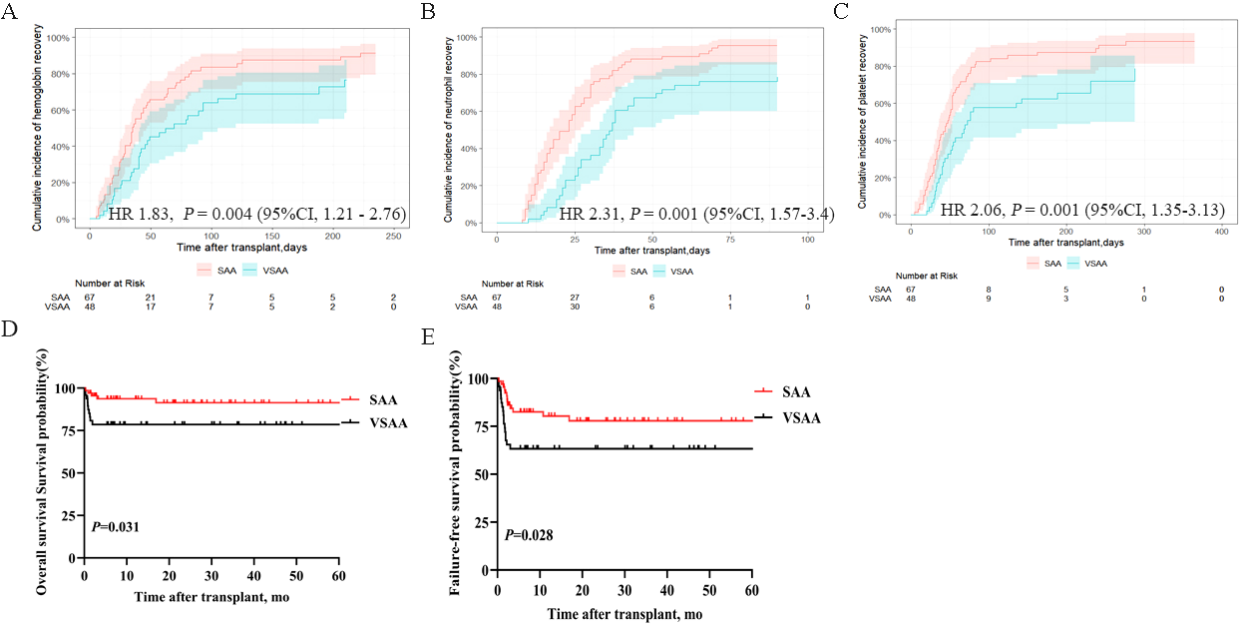
Hematopoietic recovery and survival outcomes in patients with SAA and VSAA who underwent IIST-UCB. **(A)** Cumulative incidence of Hb recovery; **(B)** Cumulative incidence of neutrophil recovery; **(C)** Cumulative incidence of PLT recovery; **(D)** OS in the two groups; **(E)** FFS in the two groups.

**Supplementary Table S1** shows the baseline demographic and disease characteristics of patients with SAA and VSAA. In the SAA group, the median time to neutrophil engraftment was 20 days (range, 8–110), while PLT engraftment was 44 days (range, 4–276l) in the SAA group. In the VSAA group, the median time to neutrophil engraftment was 28 days (range, 8–90), and time to PLT engraftment was 43 days (range, 6–288) in the VSAA group. The 250-day cumulative incidences of Hb recovery were 91.3% (95% CI, 79.4%–96.5%) in the SAA group and 76.6% (95CI, 58.4%–87.6%) in the VSAA group, with a HR of 1.83 (95%CI, 1.21–2.76, *P* = 0.004) (**Figure 3A**). The 100-day cumulative incidence of neutrophil engraftment were 95.5% (95% CI, 85.1%–98.7%) in the SAA group and 78.4% (95CI, 61.6%–88.5%) in the VSAA group, with an HR of 2.31 (95%CI, 1.57–3.4, *P* = 0.001) (**Figure 3B**). The 300-day cumulative incidence of PLT engraftment was 93.4% (95% CI, 81.3%–97.8%) in the SAA group and 78.6% (95%CI, 54.5%–90.8%) in the VSAA group, with an HR of 2.06 (95%CI, 1.35–3.13, *P* = 0.001) (**Figure 3C**). The 5-year OS rates were 91.4% ± 7.45% and 78.6% ± 11.76%, respectively (*P =* 0.028) (**Figure 3D**); the 5-year FFS rates were 77.91% ± 10.78% and 63.3% ± 13.92%, respectively (*P* = 0.028) (**Figure 3E**).

## Discussion

In the absence of an HLA-matched sibling donor, mismatched alternative stem cells serve as a salvage treatment option for patients with SAA who have failed first-line IST. Due to its low incidence of GVHD, UCB is an important stem cell source (16, 17). Advances in transplantation technology, have led to considerable improvements in treatment outcomes with IIST-UCB in recent years. To our knowledge, this is the first and largest retrospective analysis to assess the efficacy of IIST-UCB.

Early hematopoietic progenitors from UCB can survive, proliferate, differentiate, and produce hematopoietic-stimulating factors in the patient’s body for a limited time, resulting in short-term hematopoietic replacement. Shortening the neutropenic phase can reduce complications such as infection and bleeding following immunosuppression, while also improving overall efficiency. Our previous study demonstrated that IIST-UCB provided a significant survival advantage for patients with SAA compared to IST alone. The IIST-UCB group showed a slightly higher OR rate at 6 months post-transplant compared to the IST group (10). Building on previous research, this study investigated the efficacy and feasibility of IIST-UCB in patients with SAA using a larger sample size. Given the impact of relapse and graft failure on quality of survival, OS was not used as the sole evaluation endpoint. FFS may offer a more accurate assessment of post-transplantation outcomes. The OS and FFS outcomes in the IIST-UCB are consistent with previous findings from our center (10). Cytomegalovirus (CMV) infection remains a common and potentially fatal complication following HSCT (18, 19). Although immunosuppressive agents were used in the IIST-UCB group, the rate of viral infections was not persistently high, which may be attributable to the immunomodulatory properties of UCB transplantation.

Patients with SAA predominantly exhibit a state of microchimerism. Mixed chimerism can convert to microchimerism at various time points after transplantation, and a prolonged microchimerism state is still capable of supporting effective hematopoiesis in patients with SAA. In addition, human UCB is rich in hematopoietic stem cells, which can differentiate into hematopoietic and immune cells, playing an immunomodulatory role in patients and thereby reducing the incidence of GVHD. These findings suggest that IIST-UCB could be considered for patients with SAA who lack an MSD, as it is readily available without delay. Moreover, patients in the SAA group who received UCB treatment demonstrated higher hematopoietic reconstitution efficiency and OS rates compared to those in the VSAA group. This suggests IIST-UCB may be more effective in SAA than in VSAA.

Although our findings demonstrate the efficacy of IIST-UCB in treating SAA, we acknowledge several limitations associated with UCB transplantation. Adult patients often require double cord units due to insufficient cell doses in single units, which may increase the risk of GVHD. In our study, only single UCB units were used, and the median time to neutrophil engraftment was 25 days—slightly longer than that reported with other stem cell sources. This delayed hematopoietic reconstitution may partly explain the observed infection rates (EBV: 16.8%; CMV: 7.18%), as prolonged neutropenia predisposes patients to viral reactivation.

With significant advancements in graft manipulation, reduced-intensity conditioning, and GVHD prevention, haplo-HSCT has become a feasible option for patients with SAA lacking matched donors in recent years. Despite its rapid donor availability and strong engraftment potential, haplo-HSCT carries a higher risk of GVHD compared to UCB transplantation. In contrast, UCB transplantation offers specific advantages, such as quicker availability and lower rates of GVHD. Although haplo-HSCT has become more common, UCB remains a valuable option, particularly when reducing GVHD risk is a priority or when haploidentical donors are unavailable.

This study has several limitations, including its retrospective, single-center design. The extended inclusion period (2004–2024) may have introduced variability due to changes in supportive care practices and transplantation techniques over time. Importantly, the absence of a direct comparison group—such as patients receiving haploidentical transplants or other alternative donor sources—limits our ability to draw definitive conclusions about the relative efficacy of IIST-UCB compared to other available options. Furthermore, potential selection bias cannot be excluded, as patients receiving this treatment approach may have differed systematically from those pursuing other therapeutic strategies. Given these limitations, further prospective studies with larger cohorts and controlled comparisons involving other alternative donor approaches are needed to validate our findings.

## Conclusion

In conclusion, this retrospective observational study evaluated the efficacy of IIST-UCB in patients with SAA. The results suggest that IIST-UCB promotes hematopoietic recovery without increasing the risk of GVHD. The therapeutic efficacy of IIST-UCB in the SAA group was superior to that in the VSAA group. Therefore, these results suggest that this promising clinical approach warrants further investigation. In addition, these results should be confirmed in prospective controlled studies, including comparative analyses with haploidentical transplantation.

## Data Availability

The raw data supporting the conclusions of this article will be made available by the authors, without undue reservation.
