# Supplementary material for: Unveiling new horizons in severe aplastic anemia management: a two-decade study on intensive immunosuppressive therapy combined with unrelated cord blood efficacy
Source: Front Immunol. 2025 Jul 18;16:1622326. doi: 10.3389/fimmu.2025.1622326 (PMC12313501; doi:10.3389/fimmu.2025.1622326)
Supplement: Supplementary file 1 [file Table1.docx]

**Table S1. Clinical characteristics of patients**

| Characteristic | SAA (N = 67) | VSAA (N = 48) | P |
| --- | --- | --- | --- |
| Median age, years (range) | 15 (2-74) | 19 (3-64) | 0.082 |
| ≤20 years | 46 (68.66%) | 26 (54.17%) |  |
| 20–40years | 16 (23.88%) | 14 (29.17%) |  |
| ≥40 years | 5 (7.46%) | 8 (16.66%) |  |
| Patient sex, n (%) |  |  | 0.579 |
| Male | 37 (55.22%) | 29 (60.42%) |  |
| Female | 30 (44.78%) | 19 (39.58%) |  |
| SAA with PNH clone, n (%) | 3 (4.48%) | 4 (8.33%) | 0.647 |
| Median cord TNC, ×10^8^/kg (range) | 18.6 (12.37-29.64) | 15.77 (10.38-25.01) | 0.085 |
| Median cord CD34+ cells, ×10^6^  /kg (range) | 6.51 (0.96-19.91) | 7.08 (1.77-15.14) | 0.952 |
